# The Long-Lasting Effect of Multidisciplinary Interventions for Emotional and Social Loneliness in Older Community-Dwelling Individuals: A Systematic Review

**DOI:** 10.3390/nursrep14040281

**Published:** 2024-12-06

**Authors:** Georgiana Zaharia, Vanessa Ibáñez-del Valle, Omar Cauli, Silvia Corchón

**Affiliations:** 1 Department of Nursing, University of Valencia, 46010 Valencia, Spain; geza@alumni.uv.es (G.Z.); omar.cauli@uv.es (O.C.); silvia.corchon@uv.es (S.C.); 2 Frailty Research Organized Group (FROG), University of Valencia, 46010 Valencia, Spain; 3 Chair of Healthy, Active and Participative Ageing, University of Valencia, 46010 Valencia, Spain

**Keywords:** loneliness, emotional loneliness, social loneliness, social isolation, psychological effects, elderly, community-dwelling individuals

## Abstract

Background: Loneliness can occur at any age, but it is more prevalent among older adults due to the associated risk factors. Various interventions exist to improve this situation, but little is known about their long-term effects. Our aims were to determine if these interventions have long-lasting effects and for how long they can be sustained. Additionally, we aimed to analyze if the interventions carried out by volunteers affected the outcomes regarding loneliness and psychological impact. Methods: A systematic review was performed by searching the literature in the MEDLINE PubMed, SCOPUS, Web of Science, PsycINFO, and Web of Science databases for interventions focused on the lonely population. The inclusion criteria for this review were the assessment of loneliness using a validated tool, and loneliness being the primary or secondary outcome. The CASPe checklist was used to assess the risk of bias in the selected studies, and the PRISMA-ScR recommendations were followed to present and synthesize the results. Results: Thirty articles were included. The interventions identified were classified into five categories: psychosocial, technological, health promotion, physical exercise, and multicomponent interventions. Loneliness improved in 24 studies during the post-intervention analysis. Social connectivity and depressive symptoms also improved in most interventions. Long-term follow-ups were conducted with positive results in a total of 16 interventions. Depressive symptoms and social connectivity were also improved. Eight of the interventions were carried out by volunteers and showed good results regarding loneliness. Conclusions: The results obtained in this work suggested that multidisciplinary interventions can reduce loneliness, but more controlled clinical studies are needed.

## 1. Introduction

Aging is a global phenomenon affecting countries worldwide. This issue arises from increased life expectancy and low birth rates. With these projections, it is estimated that more than 25% of the population will be over 65 years old by 2050 [1]. Population aging has various economic, social, and healthcare implications. Older adults mean a smaller workforce [2] and an increased demand for healthcare services [3]. Health factors alone do not account for the emergence of loneliness. Sociodemographic and well-being factors must also be considered for a comprehensive understanding of this phenomenon.

The World Health Organization (WHO) reports that 1 in 4 older adults worldwide experience social isolation [4]. Another study indicates that the prevalence of loneliness is over 45% among older individuals [5], and this percentage increased to 59.3% in the elderly population during the COVID-19 pandemic. However, those prevalences have larger differences depending on the population studied and the country where the study was developed [6]. For example, in the Chinese population, the rate of loneliness is from 26.2% to 37.9% [7,8]; in San Francisco, the Health and Retirement Study (HRS) states that 19% of the cohort experience social isolation, and 18% suffer from loneliness. Also, HRS states that 1 in 4 adults (<51 years old) experienced both social isolation and loneliness [9].

In Spain, 20.1% of the population is over 65 years old, and this percentage is projected to increase to 30.39% by 2050. Given the country’s high life expectancy and low fertility rate, the prevalence of unwanted loneliness in Spain is significant [10]. The recently published “Barómetro de la Soledad no deseada en España 2024” estimates that 1 in 5 individuals at any age suffer from unwanted loneliness in Spain [11]. This report states that loneliness is a persistent issue, with 59% of respondents reporting that they have been experiencing loneliness at any stage of their life for more than three years [10].

Levels of loneliness change throughout one’s lifetime and can occur at any age. These levels are higher in young adulthoods and older aged individuals [12,13]. Older adults may experience a higher prevalence of loneliness because of several risk factors, such as health problems (physical, mental, or cognitive), biology and genetic factors, demography (gender differences in older adults, widowhood, or financial difficulties), and socio-environmental factors [14].

Loneliness is an unpleasant and distressing experience that can be separated into two dimensions. On the one hand, emotional loneliness is the feeling of not having people with whom to feel company or intimacy [15]. On the other, social loneliness is defined as the subjective sensation of not having enough social relationships from a quantitative point of view [16]. A distinction must be made between social loneliness and social isolation. Whereas social loneliness is a subjective feeling, social isolation is an objective feeling of deficient social relationships [17]. However, social isolation can trigger feelings of unwanted loneliness. Nevertheless, the two definitions have no bidirectional relationship [16,18].

The physiopathology related to loneliness is correlated to various factors. With regard to demographic factors such as sex, females have a higher prevalence of loneliness in society [19,20]. Environmental factors contributing to the decline of an individual’s social network’s decline include widowhood and the death of their friend(s) [21,22]. The literature shows that marital status plays a significant role in the probability of suffering from loneliness since having a partner acts as a protective factor against loneliness [22,23]. In addition, with the onset of old age, functional abilities decline, leading to a major risk of loneliness [19].

Loneliness is also a risk factor. Various studies have shown that loneliness leads to premature mortality, especially in those who live alone and do not receive visits from friends or family. These people have a higher mortality of all causes (HR 1.77 [1.61–1.95]), including cardiovascular causes (HR 2.23 (IC 95%: 1.82–2.73) [24]. Loneliness can also trigger different comorbidities in humans [25]. The presence of loneliness can also prompt psychological disorders, including a negative way outlook on life, pessimism, little acceptance, less self-care, and the adoption of unhealthy habits, such as smoking or a sedentary lifestyle. This situation can progress to more severe psychological disorders, such as anxiety, depression, and dementia.

Furthermore, and especially for old people, there is a relationship between loneliness and cognition as well as Alzheimer’s disease [26,27,28]. The greater the severity of loneliness, the more significant the impairment in cognitive function. This relationship is partially explained by the negative impact of loneliness on physical health [29]. Those factors lead to a major susceptibility and vulnerability to elder abuse or, in the worst scenarios, suicidal ideation and attempts at suicide [17]. The effects that were shown as physiological disorders are a higher risk of metabolic syndrome, high blood pressure, and cardiovascular diseases [28,29].

Loneliness can also be associated with sleep disorders, a deficit in the immune system, pro-inflammatory gene expression, functional disability, and, as mentioned before, accelerated cognitive decline [2,13,25].

All those negative health outcomes lead to a worsening of the general state of health, triggering an increasing rate of longer hospitalization and readmission, as well as an increasing frequency of visits to primary healthcare [30].

Loneliness is an important problem for people’s health, and effective interventions are needed to cope with it. Various disciplines of knowledge, including medicine, psychology, and nursing, have studied different interventions to reduce loneliness. From a psychological perspective, interventions, such as cognitive behavioral therapy, emotional support therapy, and programs to promote social skills, may be carried out [31], while nursing and medicine adopt a holistic perspective on health and well-being, providing social and emotional support and focusing on assessing needs, making home visits and contacting social care services. They can also identify risks, develop an individualized intervention plan, and provide advice and health education [32].

The strategies commonly used to cope with loneliness are building social networks or performing activities to maintain a good cognitive level. Retirement is a social phenomenon that occurs with old age and leads to declining social networks. To maintain their well-being, people may resort to other ways of being part of a new group, such as beginning a new hobby or performing voluntary work [33]. Most interventions require two essential factors: sharing a physical space and being face-to-face with people with whom we want to create a connection. However, these strategies were not implemented due to the COVID-19 pandemic. New ways of relating had to be sought in this situation, such as digitization. However, other authors argue that it is not an alternative for elderly people. These authors suggest that old people do not have the devices or the knowledge and skills needed to manage new technologies [34,35,36]. As discussed above, many interventions have been developed in recent years, but there is no consensus on which ones are the most effective. It is also important to determine the immediate effects of the interventions, but the long-term effects (understood as beneficial effects on a person’s quality of life and health that persist for a period longer than 6 months; although this term varies in the literature, as other authors consider that a long-term effect should last more than 24 months [37,38]) of multidisciplinary interventions addressing loneliness are still little known. Multidisciplinary interventions involve professionals from different disciplines and areas of expertise working together and contributing with their perspective in person-centered interventions [39,40].

## 2. Materials and Methods

A systematic review was conducted to achieve the objectives of this study, and a search strategy was designed to identify the pertinent literature. The databases searched included Medline (PubMed), Web of Science (Scopus), PsycINFO, and Web of Science (WOS). The search strategies were adapted to each database’s command language, controlled vocabulary, and appropriated search fields. A manual search for additional studies was also conducted using the snowballing technique, and reference lists of identified studies were screened for additional reviews.

### 2.1. Identifying the Research Question

The PRISMA-ScR recommendation [41] was followed for the selection process, and to evaluate the critical appraisal, we used the CASPe chart for clinical trials [42].

Following the mnemonic PICO [39], we focused and contextualized the review:

- POPULATION: Older community-dwelling individuals who are above 65 years old and who experience loneliness.
- INTERVENTION: multidisciplinary interventions addressing loneliness.
- COMPARATION: comparing to the control group.
- OUTCOME: Reduction in loneliness and associated factors.

For this, we developed the following question:

Are multidisciplinary interventions in older community-dwelling individuals aged 65 and above who experience loneliness effective in reducing loneliness compared to not implementing an intervention?

The MeSH terms used were “aged”, “dwelling individuals”, “loneliness”, and “interventions”, and all their synonyms and related terms. The search terms were also consulted with a public health librarian. The research strategy was set up using the Boolean operators AND and OR. The electronic search was performed on 29 January 2024, with a subsequent update on 27 February 2024 using the terms listed in Table 1 below.

Regarding inclusion and exclusion criteria (see Table 2), studies published in English, French, and Spanish were included to map evidence of social and emotional loneliness. A date filter was applied to select articles published within the past ten years. The age filter targeted older adults (65+ years), as defined by the MeSH term “Aged” [40], although there was no strict age restriction in the final selection of articles. Studies mentioning social and/or emotional loneliness, social isolation, and multidisciplinary interventions were chosen. No restrictions were applied regarding geographical location or methodological design. Systematic reviews, meta-analyses, and scoping reviews were initially included in order to gather more data using the snowballing technique.

Studies that only focused on mental health or on patients who were hospitalized or living in a nursing home were excluded.

### 2.2. Literature Search Methodology

Regarding inclusion and exclusion criteria (see Table 2), studies published in English, French, and Spanish were included to map evidence of social and emotional loneliness. A date filter was applied to select articles published within the past ten years. The age filter targeted older adults (65+ years) as defined by the MeSH term “Aged” [40], although there was no strict age restriction in the final selection of articles. Studies mentioning social and/or emotional loneliness, social isolation, and multidisciplinary interventions were chosen. No restrictions were applied regarding geographical location or methodological design.

### 2.3. Procedure

When determining which articles to include, duplicated studies, books, editorials, communications in conferences, or letters were removed using the RefWorks website (https://refworks.proquest.com/, accessed on 20 July 2024).

A two-step process was selected to conduct the research. The first step involved a review of the titles and abstracts by the first author (GZ) based on the relevance of the topic studied. The second step was a full-text reading assessed by GZ using the inclusion and exclusion criteria. The co-authors (SC and VI-dV) then validated the articles selected. All disagreements that arose were discussed as a group with the experts on the subject (OC). The risk of bias was assessed by the first author and co-author (GZ and SC) using the CASPe checklist.

The articles found by manual research using the snowballing techniques fulfilled all the inclusion criteria, and no exclusion criteria were included.

## 3. Results

### 3.1. Selection Process

**Figure 1:**
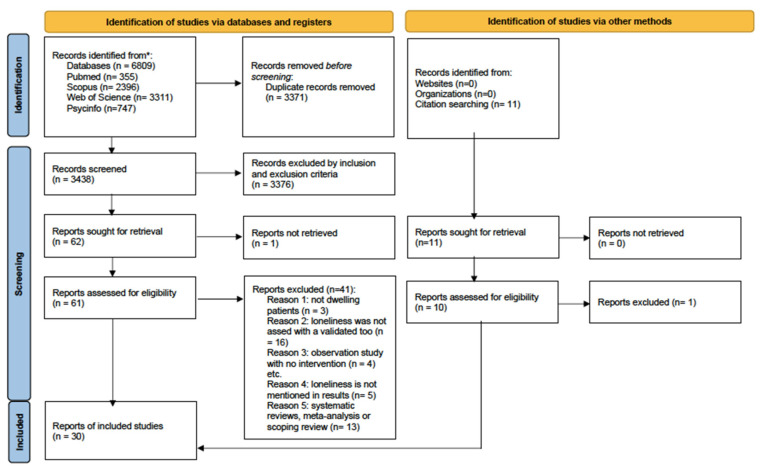
PRISMA flowchart. From: Page et al. [43]. * Consider, if feasible to do so, reporting the number of records identified from each database or register searched (rather than the total number across all databases/registers).

A total of 6809 articles were retrieved. We performed the quality assessment after eliminating duplicates and applying inclusion and exclusion criteria. A total of 30 articles were finally selected for this review (see Figure 1).

### 3.2. Critical Appraisal Assessment

The selected studies were critically reviewed using the CASPe checklist [42]. As all the results are clinical trials, we used the specific CASPe checklist for the clinical trials (see Table 3).

When interpreting the results obtained, we considered them in sections. The first section covers questions 1 to 5. Question 1 addresses whether the research question is clear and aligned with the objectives of this systematic review. All the articles have an affirmative response to this question. Most of the articles found are clinical trials but not randomized (10 out of 31), as this type of intervention is difficult to blind. In many of the articles, the intervention group and the control group consist of a waitlist. After the intervention, the roles were reversed, and the control group underwent the intervention, and vice versa. In three articles (33–35), question 4 was answered as “can’t tell”. All the questions in this section were answered with a “yes” in only five articles, indicating good internal validity with reliable and unbiased results. Four out of the five questions in the section received positive responses in 15 articles. Given that 20 of the 30 articles received between four and five positive responses, the articles selected have good internal validity and, therefore, reliable results. In the second section, questions 6, 7, and 8 addressed the external validity, i.e., the applicability of the results in other contexts. In this section, 23 of the 31 articles met the criteria of treating both intervention groups similarly and measuring and expressing the analysis of all the variables mentioned in the protocol. All the articles used a confidence interval of between 90% and 98.75% in their analysis. Finally, the third section evaluates the relevance of the results obtained. In this review, the results of four articles cannot be applied to the target population of this review. Ultimately, all articles estimate that the benefits justify the risks and costs.

Given that all the studies selected aimed to reduce loneliness using a validated scale, we estimate a low risk of bias in participant selection and result reporting.

### 3.3. Analysis of the Included Studies

A qualitative synthesis of the data from all the studies selected (n = 30) is provided in Supplementary Materials. The following data were collected: (1) reference; (2) brief description of the intervention; (3) type of intervention; (4) whether the intervention was performed by certified investigators or volunteers; (5) variables collected and results pre- and post-intervention; (6) variables collected and results during the post-intervention follow-up period. One of the 30 articles is observational [53], and 16 of them are randomized clinical trials. The results obtained also included pilot trials [44,51,59,60,63]. The rest of the selected articles are non-randomized clinical trials. The location where the intervention was carried out varied. The most prevalent country is the United States, where six interventions were conducted there [46,51,57,59,65], followed by the United Kingdom, where four interventions were conducted [47,60,67,72]. Finally, four interventions were carried out in Spain [50,52,54,74].

**Figure 2:**
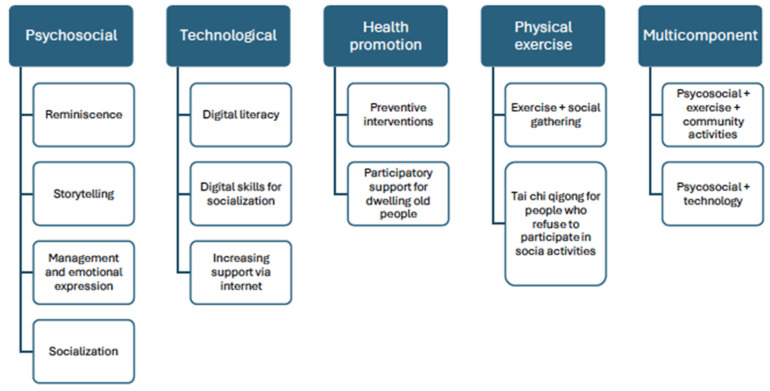
Categorization and subcategorization of the interventions included in this systematic revision.

The interventions focusing on loneliness have been classified into five types (see Figure 2).

#### 3.3.1. Psychosocial Interventions

We identified eight psychosocial interventions. The interventions carried out include the guided practice of cognitive-based compassion training (CBCT) tailored to older adults [44], eudaimonic well-being [46], psychotherapy based on the loneliness model [50], reminiscence and storytelling [57], group emotional expression [58], individual and group sessions for identifying socialization barriers [37], social prescribing to reduce loneliness [67] and emotional support via telephone [68]. These interventions aim to address psychological and social aspects to enhance personal well-being and promote socialization among individuals. All these interventions were conducted in groups, although three combined face-to-face individual sessions and group sessions [50,57,60]. Combining both formats aims to tailor each intervention to everyone’s needs. In all the articles, loneliness decreased immediately after the intervention. Moreover, loneliness is not the only measure that improves. The same applies to life satisfaction [44], connectivity [44,50,54], depressive symptoms [46,57,68], resilience [44,68], state of health, and quality of life [48,58]. The professionals who carried out these interventions were mostly psychologists and social workers, but some were performed by trained volunteers [49,60,67,68]. There was one exception [60]. A reduction in loneliness was achieved in all the interventions. Follow-up is conducted in three of the interventions. In two of them, it was conducted 6 months after the intervention ended [46,50], and in one, it was conducted 24 months after the intervention ended [49]. Loneliness declines in all the follow-ups.

#### 3.3.2. Technological Interventions

This review identified eight articles that use technology as a mitigator of loneliness. The specific interventions found include social Internet-based activities (SIBAs) that combine individual and group meetings, including in-home support and remote support via the Internet [45], PRISM software with a “friends forum” and information about municipal activities [65], weekly iPad usage sessions [51], digital literacy [55,72,73], virtual reality [59] and the online administration of behavioral therapy to improve loneliness [69]. These interventions aim to increase digital literacy and enhance technological skills so that older adults can use them as tools to boost communication and socialization. These interventions were particularly useful during the COVID-19 pandemic. One of the interventions was conducted during the lockdown [63]. In all the interventions, loneliness was reduced, except for one [51], in which no significant changes were observed. In these types of interventions, social support [55,65,72,73] and symptoms of anxiety and depression showed the biggest improvement [59,69]. Three of the interventions were performed by trained volunteers [51,55,72], but in the intervention performed by Fields et al. (2021), there were no significant changes in loneliness scores [51]. As for long-lasting effects, in the intervention using virtual reality, there was a follow-up at 2 months post-intervention, where loneliness and depression were still reduced, and sleep quality increased [59]. In the intervention conducted by Käll et al. (2020) using Internet-delivered cognitive-behavioral therapy, loneliness, depression, anxiety, and quality of life showed long-term improvement at a 24-month follow-up [69].

#### 3.3.3. Health Promotion Interventions

In this category, we found three interventions: improving the mental well-being of older people through preventive interventions related to occupation [47], participatory management intervention for group care in older people who live alone in the community [48], and weekly phone calls addressing healthcare problems [53]. These interventions aim to teach strategies for health promotion and prevention and to improve social connectivity through engagement in activities and volunteering. Loneliness improved in two of the three interventions [48,53] and the participants’ quality of life also showed improvement [48]. In this category, one of the interventions was carried out by volunteers, but no significant changes were observed in the loneliness score [53]. Finally, in the intervention conducted by Mountain et al. (2017), which aimed to improve the well-being of older adults through engagement in social activities, follow-up was conducted at 24 months and showed a significant decline in loneliness levels [47].

#### 3.3.4. Physical Exercise Interventions

Three interventions use physical exercise to cope with loneliness. One involves the practice of Tai Chi Qigong, with people who refuse to participate in social activities [70]; the second uses varied physical exercise sessions and social gatherings to increase social support [71], while the third uses physical exercise, comparing sessions via Zoom or on a website [62]. In all three interventions, loneliness improved, as well as social support. Improvements were also observed in well-being, quality of life, depressive symptoms [71], and self-esteem [70]. In the intervention using Tai Chi Qigong [70], elderly neighborhood volunteers participated in this intervention to reach the “hidden elderly”. “Hidden elderly” refers to the population who refuse to participate in social activities. The use of volunteers in this type of intervention showed improvements in loneliness levels, social support, and the participants’ quality of life. The long-lasting effects were evaluated in two interventions. One of these is the intervention performed by Chan et al. (2017), where the improvements seen post-intervention were maintained at 6 months [70]. The long-lasting effects were also measured at 9 months in another intervention, which also showed improvements in loneliness, depression, social connectivity, well-being, and quality of life [71].

#### 3.3.5. Multicomponent Interventions

This category contains five interventions [52,54,56,63,64], which combine different types of interventions. The activities included are mindfulness, yoga, walking, community visits, and a combination of technology, laughter therapy, music therapy, and relaxation, among others. Loneliness improved in all the interventions except for one [54], in which no significant changes were observed. Numerous psychological effects are associated with multimodal interventions. Depressive symptoms significantly improve [63,70], as well as the degree of dependency, cognitive level, and social support [52]. Three interventions in this category were carried out by volunteers [52,54,61]. All the interventions showed a post-intervention improvement in loneliness, except for the intervention by Hernández-Ascanio et al. (2022), although during the follow-up, they did observe improvements in loneliness and social support [54]. Finally, regarding the long-lasting effects of the intervention by Chow et al. (2019), improvements were observed in loneliness, social connectivity, self-esteem, quality of life, and mental and health status at the three-month follow-up [61].

Supplementary Materials summarizes the most important features of the interventions.

## 4. Discussion

This systematic review evaluates the effectiveness of multidisciplinary interventions to improve emotional and social loneliness in older community-dwelling individuals and the long-lasting effect of those interventions. Of the 30 articles reviewed, 24 showed a decline in levels of loneliness, while six showed no change. None of the studies showed an increase in loneliness scores. Therefore, we can state that most of the interventions studied improve loneliness scores.

### 4.1. Types of Interventions

This study established five categories of interventions: psychosocial, technological, health promotion, physical exercise, and multicomponent. Psychosocial, technological, and multicomponent interventions are the most widely used categories. Psychosocial interventions include reminiscence therapy, storytelling, management and emotional expression, and activities aimed at socialization. On the other hand, technology-based interventions include digital literacy, digital skills for socialization, and increasing support via the Internet. Multicomponent interventions, combining different workshops and activities to compensate for the weaknesses each intervention would have individually, have been gaining traction in recent years. In our systematic review, all interventions except one have demonstrated that multicomponent interventions reduce loneliness. This is the case of the study by Hernández-Ascanio et al. (2022) [54], in which a multicomponent intervention was carried out, and no significant differences were found in the social loneliness variable between the intervention and control groups in the statistical analysis. This study was not conducted by health professionals but by nursing students and volunteers with experience in the field.

On the other hand, none of the studies analyzed in this systematic review met the inclusion criteria and studied the effect of an intervention with pets on older people with unwanted loneliness. Animal-facilitated therapy (AFT), more specifically known as animal-assisted therapy (AAT) or “pet therapy”, is increasingly common in the literature [75], with a surge in recent research methodologies exploring this complementary alternative medicine (CAM) intervention. Studies like that by Hui et al. (2020) [76], in which they analyzed semi-structured interviews with 14 community-dwelling older adults who were aged 65 and above and pet owners, suggest that pet ownership may benefit community-dwelling older adults by providing companionship, giving a sense of purpose and meaning, reducing loneliness and increasing socialization. These benefits may also increase resilience against mental health disorders among older adults, which may positively influence their mental health outcomes.

### 4.2. Effects of Face-to-Face and E-Health Interventions

Of all the interventions analyzed, those of a psychosocial nature, physical exercise, and multicomponent interventions were carried out face-to-face. Face-to-face interventions have many advantages. However, face-to-face interventions may have limitations in some cases, e.g., in situations where older adults have physical conditions such as fatigue, difficulties in transporting to the intervention site, or even when there is a lack of motivation [77]. These situations can lead to low participation rates or low adherence to interventions. As a solution to this problem, interventions carried out using technological devices, such as computers or tablets, are emerging as a viable option [62,63]. E-Health emerged as an alternative response to health needs in these isolation scenarios and became more important during the COVID-19 pandemic. This phenomenon is known as e-Health. The World Health Organization (WHO) defined e-health as the cost-effective and safe use of information and communication technologies (ICT) to support health [78]. This includes telehealth, electronic health records, e-learning, telemedicine, social networking, and mHealth (mobile health, i.e., health supported by mobile devices). Studies like the one by Jung et al. (2023) [79] demonstrate that an information and communication technology (ICT) based on the Loneliness Alleviation Program (LAP) for community-dwelling older adults is effective in reducing loneliness and depression and increasing the rate of laughter among older adults.

Similarly, previous studies have shown that ICT interventions reduce loneliness [80,81,82]. However, although Internet use can benefit older people with unwanted loneliness by reducing social isolation, increasing access to services, and improving health and well-being, only a minority are online [72], and this care format has limitations. For e-Health to be effective, individuals must have an adequate level of digital literacy and skills to manage and understand these devices and the factors that affect older adults in particular. An exploratory study led by Terp et al. (2021) estimated that only 25.64% of the participants, with an average age of 81 years, were ICT users [83]. These participants revealed that factors, such as feeling that ICTs are unfamiliar, limited digital literacy, and low self-efficacy due to age-related prejudices that prevent greater uses of ICTs. Interventions are, therefore, needed to teach older people how to use new technologies and to provide resources to make telecare possible for this population group.

### 4.3. Effectiveness of Individual and Group Interventions

The literature shows that group interventions are more effective against loneliness than those conducted individually [84,85,86], and most of the studies in this review are based on group interventions. According to Masi et al. (2024), for an intervention to be effective in reducing levels of loneliness, it must meet four conditions: (1) improve social skills, (2) increase social support, (3) increase opportunities for social interaction, and (4) address maladaptive social cognition [87]. Moreover, when a person experiences unwanted loneliness, it is not by choice. The most relevant factors influencing this phenomenon are a lack of socialization and communication [88]. Taking these two factors into account, when addressing unwanted loneliness, designing group interventions that promote socialization seems crucial. Several studies support the need for group interventions, especially those related to health, as they are more effective in promoting healthy behaviors. Likewise, studies based on individual interventions have not shown the same effectiveness as group interventions.

The research by Hernández-Ascanio et al. (2022), based on individual home interventions and telephone calls made by volunteer staff and nursing students, found no significant differences between the experimental and control groups regarding variables for social isolation or loneliness [54]. Furthermore, although they observed a positive effect on improving emotional loneliness in the experimental group, this was much lower than expected. These results show that social isolation and loneliness are two multidimensional phenomena with common characteristics, but, at the same time, they have significant differences [89,90]. In the case of social isolation, interventions that enable social interactions would, therefore, be more desirable, and in the case of loneliness, psychological reorientation interventions may also be useful [91].

### 4.4. Follow-Up

In total, 16 of the 30 articles selected in this systematic review had a long-term follow-up, ranging from 16 weeks to 24 months. In most studies (7), follow-up was 6 months after the intervention, and only three studies followed up to 24 months. During follow-up, loneliness decreased in 12 studies, which was statistically significant in 10 of them.

### 4.5. Treatment/Implications for Clinical Practice

In clinical practice, healthcare professionals must be trained to detect and deal appropriately with unwanted loneliness in older people. Nurses are professionals who can detect people in situations of unwanted loneliness or at risk at an early stage. In 1987, Marjory Gordon developed the 11 Gordon’s Functional Health Patterns for use by nurses to ensure a more comprehensive nursing assessment of patients. Gordon’s functional health patterns provide a holistic model for the assessment of the family because assessment data are classified under 11 headings: health perception and health management, nutritional-metabolic, elimination, activity and exercise, sleep and rest, cognitive-perceptual, self-perception and self-concept, roles and relationships, sexuality-reproductive, coping and stress tolerance, and values and beliefs [92]. The “Roles and Relationships Pattern” explores patients’ perceptions of their role in the world. This pattern is altered if they present problems in relationships (social, family, or work) or if they feel lonely or do not have significant people (friends and family), among others. Therefore, it is necessary to encourage nurses to assess this area using appropriate scales to identify people with unwanted loneliness who need intervention. If we detect older people with unwanted loneliness, we can prescribe effective interventions with a multidisciplinary approach. Nursing is one of the professions that is more directly involved with patients, whether in hospitals or community care. As previously mentioned, nurses have the knowledge and skills to detect health issues in their early stages, such as the identification of cases of unwanted loneliness. Furthermore, due to their holistic approach, nurses are well positioned to not only select and implement the most appropriate assessment tools based on patient needs but also to coordinate and lead multidisciplinary teams for individualized interventions.

### 4.6. Strengths and Limitations

This review has certain limitations that should be considered when interpreting the results. First, the search for articles was conducted in English and Spanish, which might have excluded relevant studies published in other languages.

Second, the methodological heterogeneity of the studies selected, and the interventions analyzed made it difficult to compare the results of the studies included in this review. The heterogeneity in the interventions represents a limitation when analyzing their effects. Each type of intervention has different mechanisms of action, which complicates direct comparison and the uniform evaluation of their effectiveness in addressing loneliness. This challenge is further exacerbated when considering the cultural heterogeneity of the participating population, which is explained in more detail in the following section. In order to reduce this heterogeneity, we recommend using guidelines such as PRISMA or CONSORT for future research to improve methodological consistency.

Third, each study had its criteria for measuring the effectiveness of the interventions, as well as different inclusion criteria, so it is possible that the studies did not consider the possibility of confounding variables. Observing Supplementary Materials, we can see that three different types of scales were used to assess the degree of loneliness among participants (UCLA, De Jong Gierveld Loneliness Scale, and Short Loneliness Scale). These scales were applied in either their short or full versions. On the one hand, the use of different scales introduces variability in the results, potentially complicating the comparison between studies and compromising the overall conclusions. On the other hand, the use of short versions may omit contextual details that could enrich the understanding of loneliness within each participant’s individual experience.

Fourth, the significance of the definition of loneliness may vary depending on the cultural context in which it is developed. Cultural norms influence expectations regarding social interaction. In Western cultures, personal independence is highly valued, which means that individuals who live alone are at greater risk of experiencing loneliness. In contrast, collectivist cultures, such as those in Asian countries, promote interdependence, which could serve as a protective factor against loneliness [93,94]. In this study, the selected interventions were carried out in different cultural contexts, such as Vietnam [57], Hong Kong [70], and Spain [49], among others. Four of the selected interventions were conducted in Spain [49,50,52,54], Two of them were multicomponent interventions combining activities, such as walking [52] or health promotion [54], and the other two were psychosocial interventions [49,50]. This aspect represents a limitation, as cultural differences hinder the extrapolation of results to a general population. Each culture interprets loneliness differently based on its customs and beliefs and exhibits varying levels of receptiveness to specific types of interventions. Therefore, it would be advisable for future research to take these cultural variations into account and explore loneliness by grouping populations according to specific cultural contexts, in addition to grouping them by types of intervention.

## 5. Conclusions

Multidisciplinary interventions can improve unwanted loneliness in older community-dwelling individuals and have a lasting effect. However, further randomized clinical trials are needed to increase scientific evidence in this field. Controlled studies are needed to evaluate the efficacy of these interventions in both the short and long term.

## Figures and Tables

**Table 1:** Detailed search strategy.

SEARCH	QUERY
#1	((((((aged) OR (ageing)) OR (aging)) OR (old adult*)) OR (elderly)
#2	(((((((((Dwelling individual*) OR (community dwelling)) OR (community-dwelling)) OR (community dwelling older people)) OR (community-dwelling older people)) OR (community-dwelling older adult*)) OR (community-dwelling old adult*)) OR (community based)) OR (community-based)) OR (living in community)
#3	((((((((loneliness) OR (social loneliness)) OR (emotional loneliness)) OR (unwanted loneliness)) OR (unwilling loneliness)) OR (perce* loneliness)) OR (isolation)) OR (social isolation)) OR (unwanted social isolation)
#4	(((((((((((((intervention*) OR (program*)) OR (care*)) OR (nursing intervention*)) OR (health intervention*)) OR (multicomponent intervention*)) OR (multidimensional intervention*)) OR (multidisciplinary intervention*)) OR (interdisciplinary intervention*)) OR (multimodal intervention*)) OR (multidimensional care*)) OR (multimodal care*)) OR (complex* intervention*))
#5	#1 AND #2 AND #3 AND #4

Note: Truncated terms (e.g., adult*) were used to include possible endings.

**Table 2:** Inclusion and exclusion criteria.

INCLUSION	EXCLUSION
The loneliness assessment must be performed with a validated tool.	Studies that include institutionalized patients.
Loneliness was among the primary or secondary outcomes of the study.	Studies using patients with a dementia or any mental illness or neurodegenerative disease
Validated interventions where its effectiveness is calculated by statistical analysis.	Book reviews, editorials, communications in conferences or letters.
Type of articles: clinical trials, randomized controlled trials, meta-analysis and systematic reviews.	Studies with no ethical agreement or containing more than two negative responses using JBI tool.

**Table 3:** Critical appraisal assessment according to the CASPe checklist.

	Q1	Q2	Q3	Q4	Q5	Q6	Q7	Q8	Q9	Q10	Q11
Malaktaris, et al., 2020 [44]	Y	N	N	N	Y	N	Y	Y	Y	Y	Y
Larsson et al., 2016 [45]	Y	Y	Y	N	Y	Y	Y	Y	Y	Y	Y
Friedman et al., 2019 [46]	Y	N	N	Y	Y	U	Y	Y	Y	Y	Y
Mountain, Gail et al., 2017 [47]	Y	Y	Y	N	Y	N	Y	Y	N	Y	Y
Ristolainen, Hanna et al., 2020 [48]	Y	Y	Y	N	Y	Y	Y	Y	Y	Y	Y
Coll-Planas et al., 2015 [49]	Y	Y	U	N	Y	N	Y	Y	Y	Y	Y
Lorente-Martínez, et al., 2021 [50]	Y	N	Y	N	Y	Y	N	Y	Y	Y	Y
Fields et al., 2021 [51]	Y	Y	Y	N	Y	Y	Y	Y	Y	Y	Y
Rodríguez-Romero et al., 2021 [52]	Y	Y	Y	N	Y	Y	Y	Y	Y	Y	Y
Sandu et al., 2021 [53]	Y	N	N	N	Y	Y	Y	Y	Y	Y	Y
Hernández-Ascanio et al., 2022 [54]	Y	Y	Y	N	Y	Y	Y	Y	Y	Y	Y
Ngiam et al., 2022 [55]	Y	N	Y	N	Y	Y	Y	Y	Y	Y	Y
Ae-Ri et al., 2023 [56]	Y	Y	Y	Y	Y	Y	Y	Y	Y	Y	Y
Diwan et al., 2023 [57]	Y	N	Y	N	Y	N	Y	Y	N	Y	Y
Nazari et al., 2021 [58]	Y	Y	Y	N	Y	N	Y	Y	N	Y	Y
Knowles et al., 2017 [59]	Y	N	N	Y	Y	Y	Y	Y	Y	Y	Y
Mountain et al., 2014 [60]	Y	Y	Y	N	Y	Y	Y	Y	Y	Y	Y
Chow et al., 2019 [61]	Y	Y	Y	Y	Y	Y	Y	Y	Y	Y	Y
Granet et al., 2022 [62]	Y	Y	Y	N	Y	Y	Y	Y	Y	Y	Y
Shapira et al., 2021 [63]	Y	Y	Y	N	Y	Y	N	Y	Y	Y	Y
Bartholomaeus et al., 2019 [64]	Y	Y	Y	U	Y	Y	Y	Y	Y	Y	Y
Czaja et al., 2018 [65]	Y	Y	Y	N	Y	Y	Y	Y	Y	Y	Y
Cohen-Mansfield et al., 2018 [66]	Y	Y	Y	U	Y	Y	Y	Y	Y	Y	Y
Foster et al., 2021 [67]	Y	Y	Y	U	Y	Y	Y	Y	Y	Y	Y
Lai et al., 2020 [68]	Y	Y	Y	Y	Y	Y	Y	Y	N	Y	Y
Käll et al., 2020 [69]	Y	Y	Y	Y	Y	Y	Y	Y	Y	Y	Y
Chan et al., 2017 [70]	Y	Y	Y	Y	Y	Y	Y	Y	U	Y	Y
Levinger et al., 2020 [71]	Y	Y	Y	N	Y	Y	Y	Y	Y	Y	Y
Jones et al., 2015 [72]	Y	N	Y	N	Y	Y	Y	Y	Y	Y	Y
Rolandi et al., 2020 [73]	Y	N	Y	N	Y	Y	Y	Y	Y	Y	Y

Y = yes; U = unknown; N = no. Q1: Is the essay oriented towards a clearly defined question? Q2: Was the assignment of patients to treatments random? Q3: Were all patients who entered the study adequately accounted for until the end of the study? Was the follow-up complete? Was the study stopped early? Were the patients analyzed in the group to which they were randomly assigned? Q4: Was blinding maintained for the patients?—The clinicians?—The study staff? Q5: Were the groups similar at the beginning of the trial, in terms of other factors that could affect the outcome: age, sex, etc.? Q6: Aside from the intervention being studied, were the groups treated similarly? Q7: Is the effect of the treatment very large? What outcomes were measured? Are the outcomes measured those specified in the protocol? Q8: What is the precision of this effect? What are its confidence intervals? Q9: Can these results be applied to your environment or local population? Do you think the patients included in the trial are sufficiently similar to your patients? Q10: Were all clinically important outcomes considered? If not, how does that affect the decision to be made? Q11: Do the benefits justify the risks and costs? It is unlikely that this can be deduced from the trial, but what is your opinion?

## Supplementary Materials

The following supporting information can be downloaded at: https://www.mdpi.com/article/10.3390/nursrep14040281/s1, Table S1: Description_of_included_studies.

## Public Involvement Statement

No public involvement in any aspect of this research.

## Guidelines and Standards Statement

This manuscript was drafted against the PRISMA guideline and citation for systematic review research.
