# Supplementary material for: The Long-Lasting Effect of Multidisciplinary Interventions for Emotional and Social Loneliness in Older Community-Dwelling Individuals: A Systematic Review
Source: Nurs Rep. 2024 Dec 6;14(4):3847–63. doi: 10.3390/nursrep14040281 (PMC11676195; doi:10.3390/nursrep14040281)
Supplement: Supplementary file 1 [file nursrep-14-00281-s001.zip › nursrep-3267575-supplementary.pdf]

Table S1:

Reference	Intervention	Classification	Professional	Variables and results		Follow-up<br>(2, 3, 6. 9, 12, 24 months)
				PRE	POST	
Malaktaris, et al. 2020 [32]	Guided practice of cognitive-based compassion training (CBCT) tailored to older adults.	Psychosocial	Psychologists	Loneliness (UCLA)	↓ Loneliness (UCLA)	
				Life satisfaction (SWLS)	↑ Life Satisfaction (SWLS)*	
				Rumination (RTS)	↓ Rumination (RTS) *	
				Emotions (mDES)	↑ Emotions (mDES)	
				Empathy (TEQ)	↑ Empathy (TEQ)	
				Resilience (CD-RISC)	↑ Resilience (CD-RISC)*	
Larsson et al. 2016 [33]	Social activities carried out online (SIN).	Technological	Occupational therapists	Loneliness (UCLA)	↓ Loneliness (UCLA)*	
				Social network (SN)	↑Social network (SN)*	
				Emotions (ESI)	↓ Emotions (ESI)*	
Friedman et al. 2019 [34]	In-person sessions where you can learn about eudaimonic well-being.	Psychosocial	Nursing and social workers	Loneliness (UCLA)	↓Loneliness (UCLA)*	6 months:<br>↓ Loneliness (UCLA)*<br>↑ Wellbeing (PWB)*<br>↓ Depression (GDS)*<br>↓ Anxiety and depression (Keller symptoms*<br>↑Sleep quality (PSQI)*
				Wellbeing (PWB)	↑ Wellbeing (PWB)*	
				Depression (GDS)	↓ Depression (GDS)*	
				Anxiety and depression (Keller symptoms)	↓ Anxiety and depression (Keller symptoms *	
				Sleep quality (PSQI)	↑Sleep quality (PSQI)	
Mountain, Gail et al. 2017 [35]	Improvement the mental well-being of older people through	Health promotion	Social workers	Loneliness (JGLS)	Loneliness (JGLS)	24 months:<br>↓ Loneliness (JGLS)*<br>Mental health (SF-36)
				Mental health (SF-36)	Mental health (SF-36)	

	preventive interventions related to occupation.			Wellbeing (ONS)	Wellbeing (ONS)	Wellbeing (ONS)<br>Self-efficacy (GSE)<br>Health status (EQ-5D-3L)
				Self-efficacy (GSE)	Self-efficacy (GSE)	
				Health status (EQ-5D-3L)	Health status (EQ-5D-3L)	
Ristolainen, Hanna et al. 2020 [36]	Participatory management intervention for group care in older people who live alone in the community.	Health promotion	Healthcare professionals	Loneliness (UCLA)	↓ Loneliness (UCLA)	6 months:<br>↓Loneliness (UCLA)<br>Quality of life (QoL)<br>Trust:<br>↑Trust in other people*<br>↓Distrust in other people<br>↑Trust in the government and public authorities<br>↑Trust in public social care*<br>↓Trust in public social care<br>↑Trust in the courts*
				Quality of life (QoL)	↓Quality of life (QoL)	
				Trust:	Trust:	
				Trust in other people	↑Trust in other people*	
				Distrust in other people	↓Distrust in other people	
				Trust in the government and public authorities	↑Trust in the government and public authorities*	
				Trust in public healthcare	Trust in public healthcare	
				Trust in public social care	↑Trust in public social care*	
				Trust in the courts	Trust in the courts	
					↓Trust in the police*	
				Trust in municipal decision making	Trust in municipal decision making	
Coll-Planas et al. 2015 [37]	Coordinated action with the objective of building and strengthening the network between	Psychosocial	Volunteers, healthcare and social workers	Loneliness (JGLS)	↓Loneliness (JGLS)*	24 months:<br>↓Loneliness (JGLS)*<br>↑Social participation<br>↓Depression (GDS-5)*
				Social participation	↑Social participation*	

	health centres, senior centres and other community assets where seniors could participate in activities.			Depression (GDS-5)<br>Number of social contacts<br>Number of new activities	↓Depression (GDS-5)*<br>↑Number of social contacts*<br>↑Number of new activities*	↓Number of social contacts<br>↓Number of new activities
Lorente-Martínez, et al. 2021 [38]	Psychotherapeutic intervention based on the loneliness model.	Psychosocial	Psychologists	Loneliness (UCLA)<br>Depression (GDS)<br>Mental health (SF-36)<br>Self-efficacy (AEE)<br>Social participation (SPPI)<br>Satisfaction with intervention	↓Loneliness (UCLA)<br>Depression (GDS)<br>Mental health (SF-36)<br>↑ Self-efficacy (AEE)*<br>↑ Social participation (SPPI)<br>↑Satisfaction with intervention	6 months:<br>↓Loneliness (UCLA)<br>Depression (GDS)<br>Mental health (SF-36)<br>↓ Self-efficacy (AEE)<br>↓ Social participation (SPPI)
Fields et al., 2021 [39]	Weekly iPad lessons and internet access at home through volunteers.	Technological	Volunteers	Loneliness (UCLA)<br>Interpersonal support (ISEL)<br>Technology usage<br>Digital trust	Loneliness (UCLA)<br>↓Interpersonal support (ISEL)<br>↑Technology usage*<br>↑Digital trust	
Rodríguez-Romero et al., 2021 [40]	18 educational workshops, mindfulness, yoga, walks and visits to community gardens	Multicomponent	Nurses and volunteers	Loneliness (UCLA)<br>Autonomy (BI)<br>Mental status (SPMSQ)	↓Loneliness (UCLA)*<br>↑Autonomy (BI)<br>↑Mental status (SPMSQ)*	

				Depression (Yesavage AQ)	↓Depression (Yesavage AQ)*	
				Social support (DUKE)	↑Social support (DUKE)*	
Sandu et al., 2021 [41]	Weekly phone calls.	Health promotion	Volunteers	Loneliness (UCLA)	Loneliness (UCLA)	
				Volunteers' satisfaction	↑ Volunteers' satisfaction	
Hernández-Ascanio et al. 2022 [42]	In-person sessions on health promotion interspersed with telephone follow-up.	Multicomponent	Volunteers	Loneliness (JGLS)	Loneliness (JGLS)	6 months:<br>Loneliness (JGLS)<br>Health status (EQ-5D)<br><br>↓Social support (DUFSS)
				Health status (EQ-5D)	↑ Health status (EQ-5D)*	
				Social support (DUFSS)	↓ Social support (DUFSS)*	
Ngiam et al., 2022 [43]	Digital literacy. Participants learn digital skills and connect to social networks.	Technological	Volunteers	Loneliness (UCLA)	Loneliness (UCLA)	
				Digital literacy	↑ Digital literacy*	
				Social support (LSNS-6)	↓ Social support (LSNS-6)	
				Health status (EQ-5D-3L)	↑ Health status(EQ-5D-3L)	
				Well-being (PWS)	↓Well-being (PWS)	
Ae-Ri et al., 2023 [44]	Activity program based on laughter using technology.	Multicomponent	Nurses and social workers	Loneliness (UCLA)	↓Loneliness (UCLA)*	
				Health status (EQ-5D)	↑ Health status(EQ-5D)*	
				Depression (GDS)	↓ Depression (GDS)*	

				Laughter index scale	↑Laughter index scale*	
Diwan et al., 2023 [45]	Reminiscence and storytelling therapy at home.	Psychosocial	Community agencies providing mental health services	Short loneliness scale	↓ Short loneliness scale*	6 months:<br>Short loneliness scale<br>Depression (GDS) (PHQ)<br>Satisfaction with life
				Depression (GDS)(PHQ)	↓Depression (GDS) (PHQ)*	
				Satisfaction with life	↑Satisfaction with life*	
Nazari et al., 2021 [46]	Group therapy in which aspects such as the expression of emotions and socialization	Psychosocial	Health care professionals	Loneliness (UCLA)	↓ Loneliness (UCLA)*	
				Social participation	↑ Social participation*	
Knowles et al., 2017 [47]	Use of virtual reality as a form through avatars as a form of socialization among widowers.	Technological	Psychologists	Loneliness (UCLA)	↓ Loneliness (UCLA)*	2 months:<br>↓ Loneliness (UCLA)*<br>↓Depression (GDS)*<br>↓ Grief (GCQ)*<br>↓Bereavement (YSL)*<br>↑Sleep quality (PSQI)*
				Depression (GDS)	↓Depression (GDS)*	
				Grief (GCQ)	↓ Grief (GCQ)*	
				Bereavement (YSL)	↓Bereavement (YSL)*	
				Sleep quality (PSQI)	↑Sleep quality (PSQI)*	
Mountain et al., 2014 [48]	Telephone calls with the aim of creating a group of "phone friends". The intervention includes one-to-one sessions and group sessions.	Psychosocial	Volunteers	Loneliness (JGLS)	Loneliness (JGLS)	
				Mental health (SF-36)	↑Mental health (SF-36)*	
				Health status (EQ-5D)	↑Health Status (EQ-5D)	

				Wellbeing (ONS)	↑Wellbeing (ONS)*	
				Depression (PHQ-9)	↓Depression (PHQ-9)	
				Self-efficacy (GSE)	↑ Self-efficacy (GSE)	
Chow et al., 2019 [49]	Various sessions of Chinese cultural activities for widows focusing on the dual process.	Multicomponent	Volunteers	Loneliness (JGLS)	↓Loneliness (JGLS)*	3 months:<br>↓Loneliness(JGLS)*<br>↓Chinese Inventory of Complicated Grief<br>↓ Anxiety and depression (HADS)<br>↑Social support (ISS)
				Chinese Inventory of Complicated Grief	↓Chinese Inventory of Complicated Grief*	
				Anxiety and depression (HADS)	↓Anxiety and depression (HADS)*	
				Social support (ISS)	↑Social support (ISS)*	
Granet et al., 2022 [50]	Comparison of the effectiveness and benefits of doing physical or bine exercise via Zoom or through videos on a website.	Physical exercise	Physiotherapist	Loneliness (UCLA)	↓Loneliness (UCLA)*	6 months:<br>↓Loneliness (UCLA)*<br>↑Health status (EQ-5D)<br>12 months:<br>↓Depression and anxiety symp (K10)
				Depression and anxiety symp (K10)	↓Depression and anxiety symp (K10)*	
				Health status (EQ-5D)	↑Health status (EQ-5D)	
Shapira et al., 2021 [51]	Enable social interactions to mitigate isolation and gain digital knowledge.	Multicomponent	Social workers	Loneliness (UCLA)	↓Loneliness (UCLA)*	
				Depression (PHQ-9)	↓Depression (PHQ-9)*	
Bartholomaeus et al., 2019 [52]	Improved well-being and resilience delivered to people in the community in a	Multicomponent	Trained community workers	Loneliness (UCLA)	↓Loneliness (UCLA)*	
				Wellbeing (PERMA)	Wellbeing (PERMA)	
				Resilience (BRS)	Resilience (BRS)	

	group and to carers in another group.			Optimism (10-LOTR)	Optimism (10-LOTS)	
Czaja et al., 2018 [53]	Increasing support and isolation using PRISM, a computer program	Technological	Psychiatrists and psychologists	Loneliness (UCLA)	↓Loneliness (UCLA)*	
				Friendship Scale	↑Friendship Scale*	
				Social support (LSNS-6)	↑Social support (LSNS-6)*	
				Quality of life (QoL)	↑Quality of life (QoL)*	
				Health status (SF-36)	↑Health status (SF-36)*	
				Att. Tecno	↑Att. Tecno	
Cohen-Mansfield et al., 2018 [54]	Identification of the barriers that increase loneliness and addressing them through individual and/or group sessions.	Psychosocial	Social workers	Loneliness (UCLA)	↓Loneliness (UCLA)*	3 months:<br>↓Loneliness (UCLA)*<br>Frequency of loneliness<br>Severity of loneliness<br>Mental health (MMTSE)
				Frequency of loneliness	Frequency of loneliness	
				Severity of loneliness	Severity of loneliness	
				Mental health (MMTSE)	Mental health (MMTSE)	
Foster et al., 2021 [55]	Prescription of social activities to reduce loneliness	Psychosocial	Volunteers	Loneliness (UCLA)	↓Loneliness (UCLA)*	3 months:<br>↓Loneliness (UCLA)*<br>↑ Mental wellbeing (SWEMWBS)
				Mental wellbeing (SWEMWBS)	↑ Mental wellbeing (SWEMWBS)	
Lai et al., 2020 [56]	Intervention in a Chinese community in which telephone calls and home visits are made with the aim of providing emotional support.	Psychosocial	Volunteers	Loneliness (JGLS)	↓Loneliness (JGLS)*	
				Social support (LSNS-6)	Social support (LSNS-6)	
				Barriers to socialization (Keele)	↓ Barriers to socialization (Keele)	

				Life satisfaction	↑Life satisfaction	
				Happiness	↑Happiness	
				Depression (GDS)	↓Depression (GDS)	
				Resilience (CD-RISC)	↑Resilience (CD-RISC)*	
Käll et al., 2020 [57]	Internet-delivered intervention based on cognitive-behavioural therapy to improve loneliness.	Technological	Psychologists	Loneliness (UCLA)	↓Loneliness (UCLA)*	24 months:<br>↓Loneliness(UCLA)*<br>↓Anxiety (SIAS)*<br>↑ Quality of life (BBQoLQ)*
				Anxiety (SIAS)	↓Anxiety (SIAS)*	
				Quality of life (BBQoLQ)	↑Quality of life (BBQoLQ)*	
Chan et al., 2017 [58]	Tai chi qigong practice with the aim of improving social support and well-being of isolated older adults who refuse to participate in social activities.	Physical exercise	Volunteers	Loneliness (JGLS)	↓Loneliness (JGLS)	6 months:<br>↓Loneliness (JGLS)*<br>↑Social support (LSNS-6)*<br>↑Social Support (RSSQ)*<br>↑Mental health (SF-12)
				Social support (LSNS-6)	↑Social support (LSNS-6)*	
				Social Support (RSSQ)	↑Social Support (RSSQ)*	
				Mental health (SF-12)	↑ Mental health (SF-12)*	
Levinger et al., 2020 [59]	12 weeks of physical activity ending with a social gathering to increase social support.	Physical exercise	Physiotherapists	Loneliness (UCLA)	↓Loneliness (UCLA)*	9 months:<br>Loneliness (UCLA)<br>↑ Comm.Healthy act (CHAMPS)*<br>↑ Health status (EQ-5D-5L)*<br>↑Wellbeing (WHOS-5)<br>Depression (GDS)<br>Falls efficacy (FES-I)
				Comm. Healthy act (CHAMPS)	↑ Comm. Healthy ac (CHAMPS)*	
				Health status (EQ-5D-5L)	↑ Health status (EQ-5D-5L)*	
				Wellbeing (WHOS-5)	↑Wellbeing (WHOS-5)*	

				Depression (GDS)	↓Depression (GDS)*	↓Fall risk (FROP-COM)*
				Falls efficacy (FES-I)	↓ Falls efficacy (FES-I)*	
				Fall risk (FROP-COM)	↓Fall risk (FROP-COM)*	
Jones et al., 2015 [60]	The objective of this intervention is to evaluate the impact that the internet has on social connectivity, loneliness, etc., through volunteers and see what effect it has on them.	Technological	Volunteers	Loneliness (JGLS)	↓Loneliness (JGLS)*	
				Social support (LBNS-6)	↑Social support (LBNS-6)*	
				Mental wellbeing	↑Mental wellbeing*	
				Life satisfaction (SWEMWBS)	↑Life satisfaction (SWEMWBS)*	
				E-health readiness (PERQ)	↑E-health readiness (PERQ)*	
Rolandi et al., 2020 [61]	Intervention during pandemic with older people previously trained for "Social Networking Sites".	Technological	Not specified	Loneliness (UCLA)	↓Loneliness (UCLA)*	12 months:<br>Loneliness (UCLA)<br>↓Social support (LSNS-6)*
				Social support (LSNS-6)	↓Social support (LSNS-6)*	

↑: increase
↓: decrease
*: statistical significance
Comm. Healthy act: Community healthy activities
Att. Tecno: attitudes towards technology
UCLA: University of California, Los Angeles (UCLA) Loneliness Scale
SWLS: Satisfaction with life
RTS: Ruminative thought style questionnaire

mDES: positive and negative emotions
TEQ: Toronto Empathy questionnaire
CD-RISC: Connor-Davidson Resilience scale
SN: social network online and offline
ESI: calidad de las skills sociales cuando se participa en actividades sociales
PWB: Ryff Psychological well-being
GDS: Geriatric depression scale
Keller Symptoms questionnaire: Anxiety and depression
PSQI: Pittsburg quality of sleep index
JGLS: The Jong-Gierveld Loneliness Scale
SF-36: Short Form (36) Health Instrument (SF-36) mental health (MH) dimension
ONS: Office for National Statistics Well-being
GSE: General self-efficacy scale
EQ-5D-3L: health status EuroQol 5-Dimension 3-Level
QoL: Quality of life
AEE: Self-efficacy in Ageing Scale
SPPI: Subjective Social Participation Index
ISEL: Interpersonal support evaluation list
BI: Barthel index
SPMSQ: Pfeiffer test
Yesavage AQ: Yasevage abbreviated questionnaire for depression
DUKE: perceived social support evaluated by the Duke-UNC-11 Functional Social Support Questionnaire
DUFSS: Functional Social Support Questionnaire
LSNS-6: Lubben Social Network Scale
PWS: Personal Wellbeing Score
EQ-5D: EuroQol-5dimension
GCQ: Grief Cognitions Questionnaire
YSL: The Yearning in Situations of Loss scale for bereavement
HADS: Chinese version of the Hospital Anxiety and Depression Scale
ISS: Inventory of Social Support
K10: Kessler Psychological Distress Scale

PERMA: Wellbeing was measured using the 23-item PERMA Profiler
BRS: six-item Brief Resilience Scale
10-LOTR: 10-item Life Orientation Test-Revised
MMTSE: Mini-mental state examination
SWEMWBS: Short Warwick-Edinburgh Mental Well-being Scale
Keele assessment of participation: barriers to social participation
SIAS: Social interaction anxiety scale
BBQoLQ: Brunnsviken Brief Quality of Life Questionnaire
RSSQ: Revised social support questionnaire
CHAMPS: Community healthy activities model program for seniors
WHOS-5: World Health Organization Wellbeing Questionnaire
FES-I: the short falls efficacy scale international questionnaire
FROP-COM: The fall risk for older people in the community
PERQ: personal e Health Readiness questionnaire
